# Novel C-terminal HBx truncation (G135) in genotype D1 HBV: predicted effects on oncogenic signaling

**DOI:** 10.1186/s13027-026-00730-1

**Published:** 2026-01-28

**Authors:** Kamyar Mazloum Jalali, Hamidreza Mollaei, Chiman Karami, Elahe Mosayebnejad Roudbaneh

**Affiliations:** 1 Department of Microbiology and Medical Virology, School of Medicine, Kerman University of Medical Sciences, Kerman, Iran; 2 Department of Microbiology, Parasitology and Immunology, School of Medicine, Ardabil University of Medical Sciences, Ardabil, Iran; 3 Department of Nursing, School of Allied Medical Sciences, Guilan University of Medical Sciences, Rasht, Iran

**Keywords:** HBV, HBx protein, Mutational analysis, Genotype D1, C-terminal truncation, HCC

## Abstract

**Background & aims:**

Hepatitis B virus X protein (HBx) is a multifunctional viral regulator implicated in hepatocellular carcinoma (HCC). We aimed to characterize HBx mutations in liver tissue samples from Iranian patients with HCC and explore their potential structural and functional implications.

**Methods:**

Liver tissue samples from 10 patients with HCC and chronic HBV infection were analyzed using high-resolution melting (HRM) and Sanger sequencing. Detected variants were assessed by phylogenetic analysis, 3D protein modeling, and STRING-based protein–protein interaction (PPI) mapping. Correlations with clinical and biochemical parameters were examined.

**Results:**

Multiple HBx mutations were identified, including a novel nonsense mutation at codon 135 (G135*), resulting in C-terminal truncation. Additional substitutions were found in the H-box (V88A, K94E, A100T), BH3-like motif (L113P, Q116R, E122K, A126T), and CCCH zinc-binding domain (C61S, C69R). Structural modeling predicted that these variants may potentially disrupt DDB1 and Bcl-2 interactions, as well as zinc coordination. STRING analysis suggested altered HBx connectivity with TP53, BAX, TNF, IL6, and CREB1, potentially affecting apoptosis, transcriptional regulation, and inflammatory signaling. Phylogenetic analysis confirmed that variants clustered within genotype D1, indicating intra-genotypic adaptation. Clinically, higher viral loads and HBeAg positivity were associated with elevated liver injury markers, consistent with the predicted functional impacts of these variants.

**Conclusions:**

HBx mutations in genotype D1, particularly the novel C-terminal truncation, may potentially influence viral persistence, apoptotic resistance, and oncogenic signaling in HCC, pending experimental validation. These findings highlight intra-genotypic HBx diversity as a potential biomarker and therapeutic target for HBV-related liver cancer.

**Clinical trial number:**

Not applicable.

**Graphical abstract:**

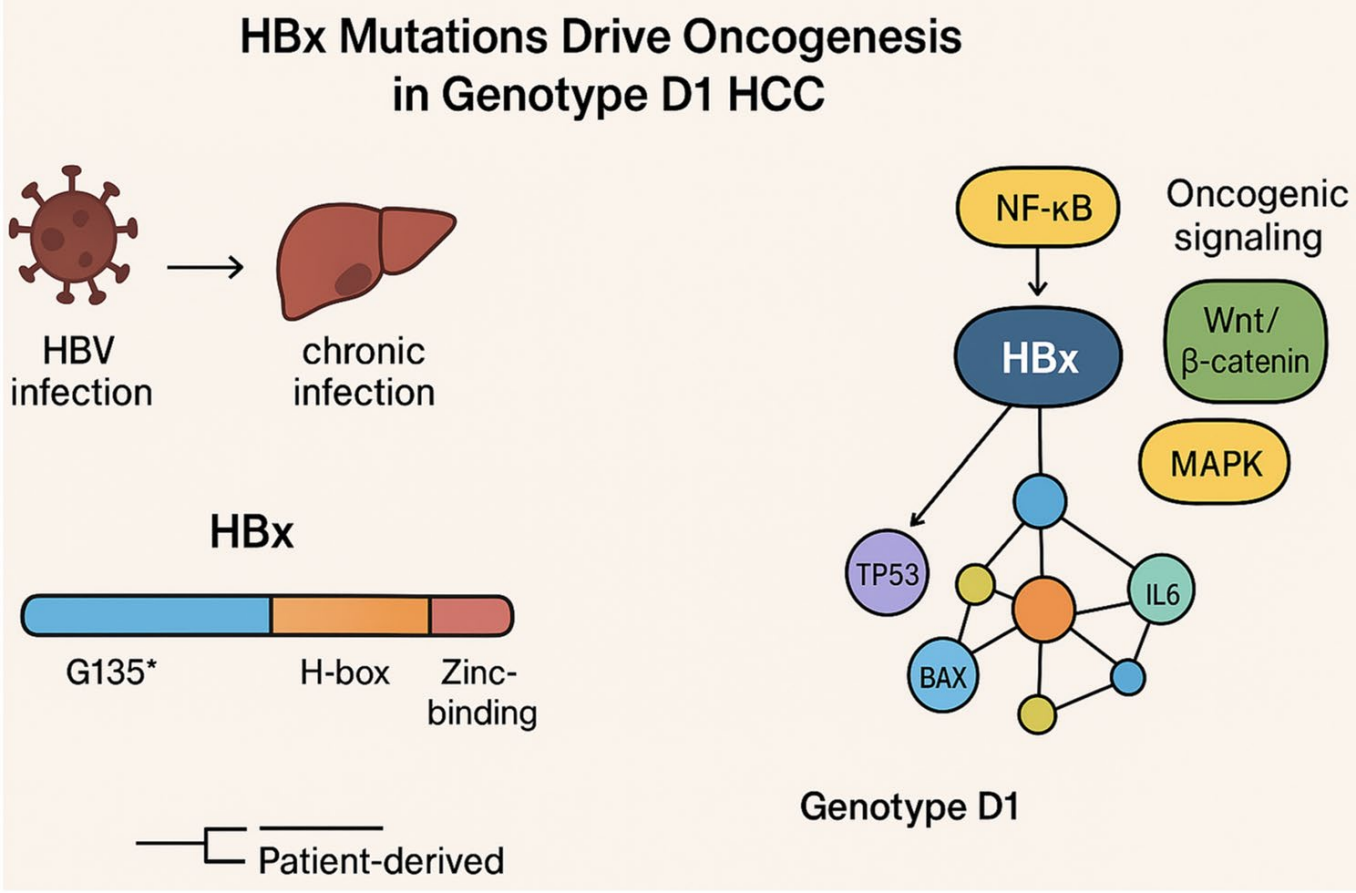

## Introduction

Hepatitis B virus (HBV) infection remains a major global health concern, with chronic infection predisposing patients to severe liver diseases including cirrhosis and HCC. The HBx plays a pivotal role in viral replication, transcriptional regulation, and modulation of host cellular pathways [1]. Accumulating evidence indicates that mutations in HBx can influence its interaction with host factors such as DDB1, p53, and apoptotic regulators, thereby contributing to hepatocarcinogenesis [2]. Previous studies have suggested that functional domains of HBx — including the H-box, BH3-like motif, and zinc-binding CCCH motif — may harbor recurrent mutations relevant to disease progression [3]. However, the mutational landscape of HBx in HCC patients from specific regional cohorts remains poorly characterized. A comprehensive analysis of these mutations is crucial for understanding the mechanistic link between viral genetics and HCC pathogenesis. To address this gap, we employed HRM analysis to detect and characterize mutations within the HBx region of pooled serum samples from HCC patients in Kerman, Iran. Earlier studies conducted on Iranian HBV-infected patients, including cohorts from Kerman, have reported diverse mutations within the Enhancer II/HBx regulatory region. These alterations were shown to affect viral promoter activity and may modulate HBV replication efficiency, underscoring the functional significance of this genomic hotspot in disease pathogenesis [4]. Key findings were further validated by Sanger sequencing to ensure accuracy and to rule out sequencing artifacts. Subsequently, the structural and functional implications of identified mutations were assessed through 3D protein modeling and STRING protein–protein interaction network analysis, providing insights into potential mechanisms of HBx-driven oncogenesis. This study aimed to delineate the population-level mutational profile of HBx in HCC patients and highlight recurrent, functionally relevant mutations — including novel variants — that may contribute to disease progression and represent potential therapeutic targets.

## Materials and methods

### Patient samples and ethical approval

This retrospective study utilized archived liver tissue samples from patients diagnosed with HCC in the context of chronic HBV infection, collected at Kerman University of Medical Sciences, Iran. All samples were fully anonymized prior to analysis. Ethical approval was granted by the Ethics Committee of Kerman University of Medical Sciences (Approval ID: IR.KMU.REC.96000733). Given the retrospective and anonymized nature of the study, the requirement for written informed consent was waived in accordance with institutional and national regulations, and with the Declaration of Helsinki.

### Clinical and laboratory assessment

Demographic and clinical data, including age, sex, HBV viral load, HBeAg status, and liver enzyme levels such as alkaline phosphatase (ALK-P), serum glutamic-oxaloacetic transaminase (SGOT), and serum glutamic-pyruvic transaminase (SGPT), were extracted from medical records corresponding to the time of liver biopsy. HBV viral load was quantified using real-time PCR (IU/mL), while liver enzyme measurements were performed using an automated analyzer (Hitachi, Japan). HBeAg status was determined via commercial ELISA kits according to the manufacturer’s instructions.

### DNA extraction, PCR amplification, and HRM screening

Genomic DNA was extracted from liver tissue samples using a commercially available kit (e.g., Qiagen DNeasy Blood & Tissue Kit) following the manufacturer’s protocol. DNA integrity and concentration were assessed using spectrophotometry. A total of 50 liver tissue samples were included for PCR-based screening of HBx sequence variations. Initial detection was performed using PCR amplification combined with high-resolution melting (HRM) analysis on a Rotor-Gene Q platform (Qiagen, Germany) with SYBR Green I chemistry. Melting dynamics were interpreted using Rotor-Gene analytical software to identify samples with potential sequence variants. Two primer sets were employed: one optimized for HRM detection (168 bp amplicon) and another covering the full-length HBx coding sequence for Sanger sequencing (506 bp). PCR reactions (25 µL) contained 50 ng of template DNA, 1× PCR buffer, 2 mM MgCl₂, 200 µM of each dNTP, 0.4 µM of forward and reverse primers, and 1 U Taq DNA polymerase (CinnaGen, Iran). Primer specificity was verified in silico using NCBI Primer-BLAST. Primer sequences and amplicon sizes are detailed in Table 1.

**Table 1:** Primer sequences used for HBx gene detection and sequencing

Name	Real-time PCR primers	Location	Product size (bp)
HBXF:	GTCTGTGCCTTCTCATCTG	265–284	168 bp*
HBXR:	GTTCACGGTGGTCTCCAT	433 − 415	
Name	**Sequencing Primers**	**Location**	506 bp
HBXSF:	ATGGCTGCTAGGCTGTGCT	-19-0	
HBXSR:	GGCAGAGGTGAAAAAGTTGC	487 − 465	

*The 168 bp product was used for initial HBx detection; the 506 bp product covered the full-length coding sequence and was used for mutation analysis

### High-Resolution melting (HRM) analysis

HRM analysis was conducted to detect potential HBx sequence variations. Amplified products were subjected to gradual denaturation, and melting profiles were analyzed to distinguish samples with altered nucleotide composition. Ten representative samples exhibiting distinct melting behaviors were selected for Sanger sequencing validation.

### Sanger sequencing validation

Pooled PCR products from liver tissue DNA were sequenced using the Sanger method. Resulting sequences were aligned against the HBx reference sequence (GenBank accession: U95551.1) using Clustal Omega. Functional hotspot mutations were annotated at both nucleotide and amino acid levels.

### Phylogenetic analysis

HBx consensus sequences derived from liver tissue samples were aligned with reference HBV sequences from GenBank. Phylogenetic reconstruction was performed using MEGA X software via the neighbor-joining method with 1000 bootstrap replicates to determine evolutionary relationships and confirm genotype identity.

### 3D protein modeling

Consensus and mutated HBx amino acid sequences were modeled in three dimensions using Swiss-Model. Structural validation and visualization were performed with PyMOL and Chimera. Mutations in functional domains, including the C-terminal, H-box, and BH3-like motif, were analyzed for potential structural and functional impacts.

### STRING protein–protein interaction analysis

The functional consequences of HBx mutations were further assessed using STRING v11.5. Protein–protein interaction networks predicted interactions with host factors, highlighting pathways potentially altered by the identified HBx variants.

### Statistical analysis

Continuous variables are presented as mean ± SD, median, and range; categorical variables as frequencies and percentages. HBV viral load values were log10-transformed. Differences between HBeAg-positive and -negative groups were evaluated using Fisher’s exact test for categorical data and Mann–Whitney U test for continuous variables. Correlations were assessed via Spearman’s rank coefficient. Statistical analyses were conducted in R (v4.3.1), with significance set at *P* < 0.05.

## Results

### Baseline characteristics

The study cohort consisted of 10 patients (6 males and 4 females) with a mean age of 44.6 years (range: 35–57 years). The median HBV viral load was 25,000 IU/mL, (rang, 350 -5,000,000 IU/mL). The mean ALK-P, SGOT, and SGPT levels were 546 U/L, 198 U/L, and 186 U/L, respectively (Table 2). Seven patients (70%) were HBeAg-positive.

**Table 2:** Baseline demographic and clinical characteristics of the study cohort. Demographic and laboratory findings of 10 pooled HCC patients with chronic HBV infection from Kerman province. Data include age, sex, HBV viral load, serum biochemical parameters (ALK-P, SGOT, SGPT), and hbeag status at enrollment

Patient ID	Age	Sex	HBV Load (IU/mL)	ALK-*P* (U/L)	SGOT (U/L)	SGPT (U/L)	HBeAg Status
Kerman_10	52	Male	350	321	35	36	Negative
Kerman_12	35	Female	650	222	42	47	Positive
Kerman_18	36	Male	5400	654	254	223	Positive
Kerman_20	46	Male	10,000	911	410	530	Positive
Kerman_25	51	Female	25,000	842	250	513	Positive
Kerman_26	42	Male	250,000	626	125	135	Positive
Kerman_28	44	Female	1100	215	98	96	Negative
Kerman_34	57	Male	6500	216	95	120	Negative
Kerman_44	39	Male	800,000	549	365	421	Positive
Kerman_47	55	Female	5,000,000	542	230	250	Positive

### Comparison of HBeAg-positive and negative groups

Patients with positive HBeAg status tended to present with higher HBV viral loads than those with negative status (median log10 viral load: 4.40 vs. 3.04 IU/mL, *P* = 0.117). No statistically significant differences were observed in the ALK-P, SGOT, or SGPT levels between the two groups (all *P* > 0.05). Comparisons between HBeAg-positive and negative patients were performed using the Mann–Whitney U test for continuous variables and Fisher’s exact test for categorical variables.

### Correlation analysis

**Table 3:** Summary of clinical and virological parameters in the study population

Variable	Mean	Median	Min	Max
Age (years)	44.6	44	35	57
HBV Viral Load (IU/mL)	807,125	25,000	350	5,000,000
ALK-P (U/L)	546	549	215	911
SGOT (U/L)	198	153	35	513
SGPT (U/L)	186	135	36	530

Continuous variables are presented as the mean ± standard deviation (SD) and median (range) in Table 3. Categorical variables are expressed as numbers (percentages). HBV viral load values were log10-transformed prior to statistical analysis. Spearman’s correlation analysis revealed a moderate positive correlation between HBV viral load and SGOT levels (*r* = 0.47, *P* = 0.11), and between HBV viral load and SGPT levels (*r* = 0.42, *P* = 0.15). Although these correlations did not reach statistical significance owing to the limited sample size, the trends suggest a potential association between viral replication and hepatocellular injury. Age was not significantly correlated with HBV viral load or liver enzyme levels (all *P* > 0.2).

**Fig. 1:**
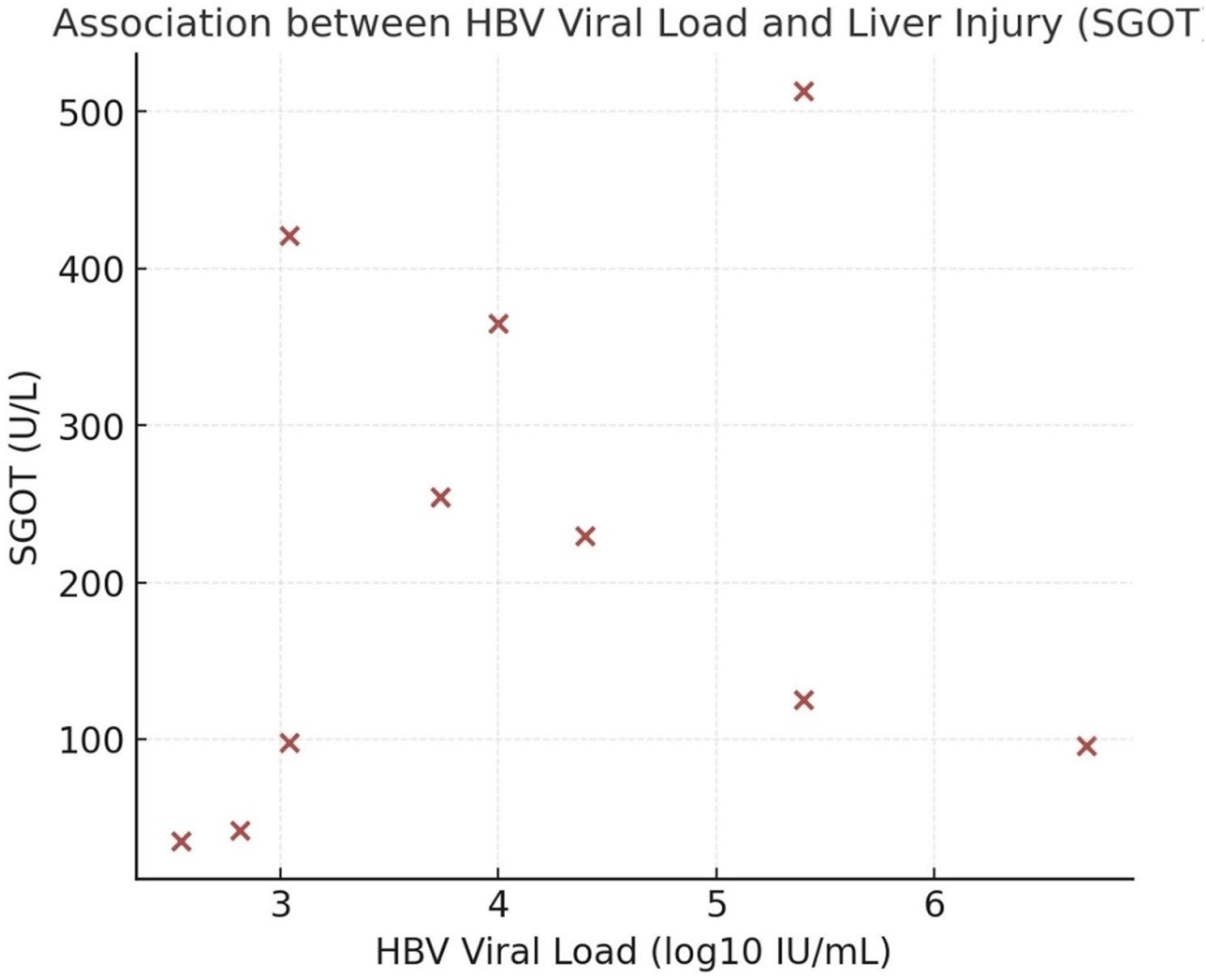
Association between HBV viral load and liver injury

Scatter plot showing the relationship between log10-transformed HBV viral load and SGOT levels in HCC patients (*n* = 10). A positive trend was observed, suggesting that increased HBV replication may be associated with increased hepatocellular injury. Scatter plot analysis demonstrated a positive trend between HBV viral load (log10 IU/mL) and SGOT levels (Fig. 1). Patients with higher viral loads tended to present elevated aminotransferase level, reflecting increased hepatocellular damage. Although this correlation was not statistically significant (Spearman *r* = 0.47, *P* = 0.11), the overall pattern suggests that enhanced viral replication may contribute to liver injury in patients with HCC.

Taken together, these findings indicate that patients with HCC active HBV replication, as reflected by higher viral loads and HBeAg positivity, exhibit a tendency toward elevated aminotransferase levels, consistent with enhanced liver injury. However, given the pooled sample size and variability in viral loads, these results should be interpreted with caution.

### High resolution melting (HRM) analysis

**Fig. 2:**
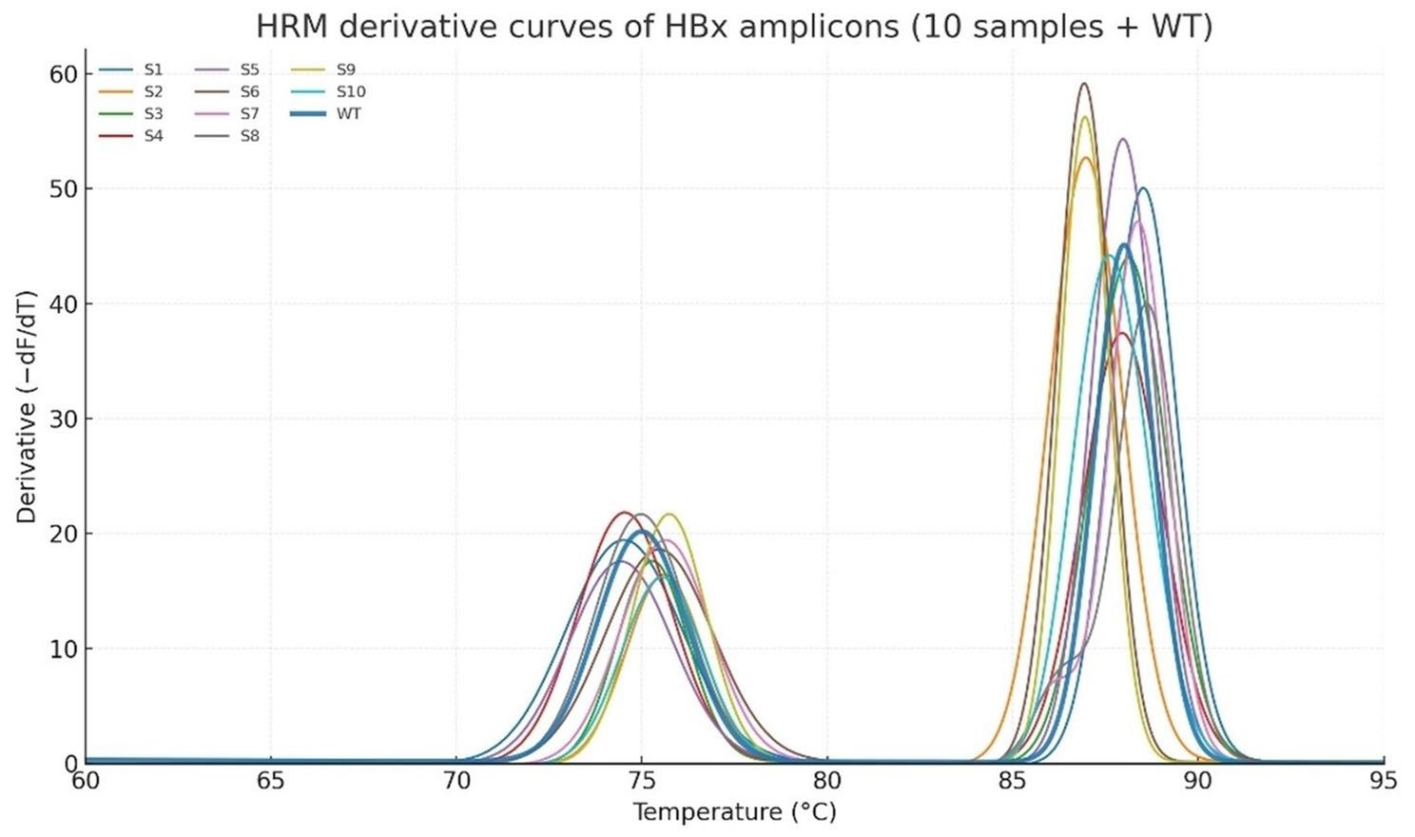
High-resolution melting (HRM) analysis of HBx amplicons

Derivative melting curves (top panel) for ten independent samples (S1–S10) compared to the wild-type (WT) control demonstrated distinct shifts in melting domains, indicating sequence heterogeneity within HBx. The difference plot relative to WT (bottom panel) clearly separated variant clusters from the wild-type, highlighting HRM as a sensitive tool for detecting minor sequence alterations in pooled HBV samples. High-resolution melting analysis of ten independent HBx-positive samples, compared with the wild-type (WT) control, revealed reproducible and distinct melting profiles (Fig. 2). All the samples exhibited a biphasic melting pattern with an early transition domain at ~ 75 °C and a major high-temperature transition ranging between 87 and − 89 °C. Compared to the WT curve, multiple samples demonstrated measurable melting shifts (ΔTm ≈ 0.3–1.2 °C) and curve-shape deviations, including broadened peaks and secondary shoulders, suggesting an underlying nucleotide heterogeneity.

**Fig. 3:**
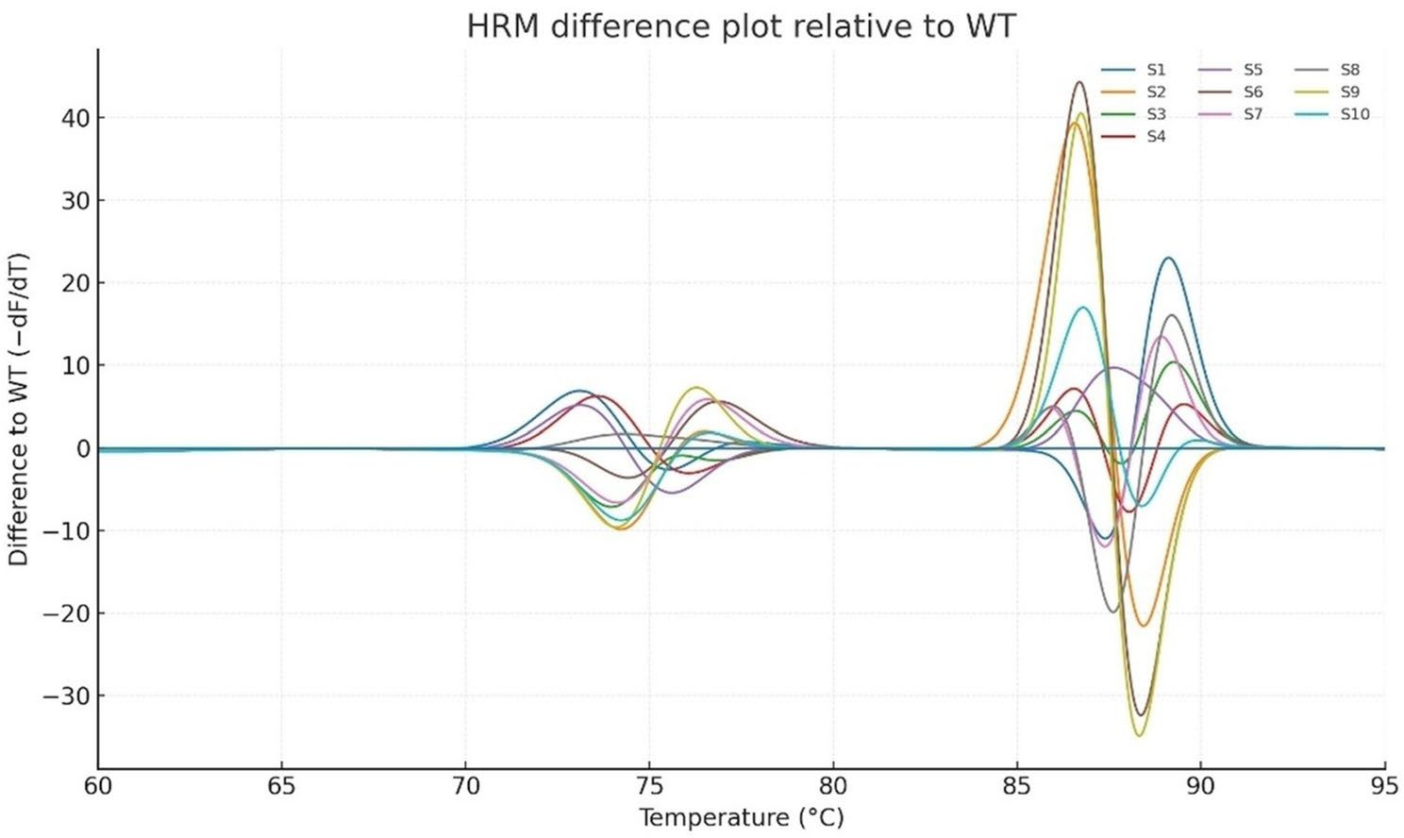
Difference plots relative to WT amplified these variations, enabling clearer discrimination of samples with subtle Tm shifts and altered curve morphology (notably S3, S5, S7, and S9). Together, the derivative and difference analyses highlight HRM as a sensitive method for detecting HBx quasispecies diversity in HCC with chronic HBV infection

Difference plots (Fig. 3) relative to the WT control further resolved these alterations by amplifying subtle curve_shape changes. Samples S3 (Kerman_18), S5 (Kerman_25), S7 (Kerman_28), and S9 (Kerman_44) showed the most pronounced deviations, clustering apart from the WT and other samples, consistent with the accumulation of point mutations or mixed viral populations. The concordance between HRM profiles and mutational heatmap analysis underscores HRM as a rapid, high-throughput tool for detecting HBx quasispecies variability in chronic HBV infections. Taken together, the derivative melting curves and difference plots consistently demonstrated that HRM is highly sensitive for detecting minor sequence alterations within the HBx. These findings are in line with the mutational heatmap and structural analyses, supporting the role of HRM as a rapid, high-throughput pre-screening tool prior to confirmatory sequencing and functional assays.

### Mutations detect in HBx

In total, we identified multiple amino-acid substitutions distributed across several functional domains of the HBx protein (Table 4). Variants located in the N-terminal regulatory region (S19A, K23R, P38T, R47Q) were positioned within segments implicated in early transcriptional modulation, although no functional effect can be concluded from sequence data alone. Additional mutations such as T55I, C61S, and C69R mapped to the transactivation/mitochondrial interaction region, a segment known to influence HBx subcellular behavior. A cluster of substitutions was detected near or within the H-box (DDB1-binding domain), including V88A, K94E, and A100T, placing these changes in a region essential for host-factor engagement. Mutations in the p53-interaction zone (D102N, L113P, Q116R, E122K, A126T) were also observed, spanning a region frequently involved in HBx–host signaling interfaces. Notably, a novel premature stop-gain mutation (G135*) was detected, resulting in the loss of the downstream C-terminal sequence, including residues C137 and H139. This truncation removes part of the regulatory interface that is normally retained in full-length HBx. While the biological relevance of this truncation cannot be established without experimental studies, its presence provides the structural basis for the subsequent in-silico analyses. Overall, the mutation pattern observed across the patient-derived HBx sequence spans multiple functional elements, and the positional mapping of these variations offers a structural framework for understanding their potential relevance in HBV-related HCC.

**Table 4:** Summary of HBx mutations identified in HCC (Kerman cohort)

Position	aa change	Domain	Motif	Functional Implication
19	S19A	N-terminal regulatory (1–50)	Spindlin1-binding (2–21)	May affect HBx–Spindlin1 interaction, alters transcriptional regulation
23	K23R	N-terminal regulatory	—	Charge shift; may alter early regulatory interactions
38	P38T	N-terminal regulatory	—	Proline substitution may disrupt local folding
47	R47Q	N-terminal regulatory	—	Alters polarity; may affect host protein binding
55	T55I	Transactivation domain (51–140)	Start of mitochondrial targeting region (55–72)	May affect HBx mitochondrial localization
61	C61S	Transactivation	Mitochondrial targeting region (55–72)	May affect HBx-induced mitochondrial signaling
69	C69R	Transactivation	Mitochondrial targeting region (55–72)	Loss of cysteine may disturb mitochondrial interactions
76	L76F	Transactivation	Near p53-binding region (70–120)	May alter HBx–p53 interaction surface
88	V88A	Transactivation	H-box / DDB1-binding (88–100)	May reduce HBx–DDB1 binding; affects viral replication
94	K94E	Transactivation	H-box / DDB1-binding	Charge reversal; strong effect on DDB1 binding
100	A100T	Transactivation	H-box boundary	Alters stability of DDB1-binding motif
102	D102N	Transactivation	p53-binding region (70–120)	May weaken HBx–p53 interaction
113	L113P	Transactivation	BH3-like motif (110–135)	Distorts α-helix, affects apoptosis signaling
116	Q116R	Transactivation	BH3-like motif	Charge alteration; may disrupt apoptotic regulation
122	E122K	Transactivation	BH3-like motif	Polarity change; potential oncogenic enhancement
126	A126T	Transactivation	BH3-like motif	Alters hydrophobic packing in BH3-like region
135	G135*	**Transactivation domain (still inside 51–140)**	**Terminal end of BH3-like motif (110–135)**	**Premature stop; truncates entire C-terminal tail & downstream interaction sites**
137 / 139	“Lost”	Not valid	Not CCCH motif	These residues are lost due to truncation, but they are *not* zinc-finger residues

**Fig. 4:**
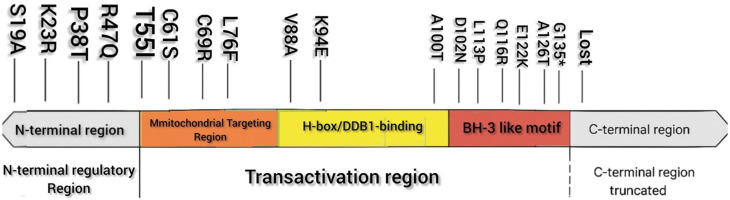
Schematic representation of HBx functional domains and the location of detected mutations

A linear map of the HBx protein is shown, illustrating the position of all amino-acid substitutions identified in the present study. Mutations cluster within multiple functional regions, including the N-terminal regulatory segment, the transactivation region, the mitochondrial targeting region, the H-box/DDB1-binding domain, and the BH3-like motif. The novel stop-gain mutation G135* results in the loss of the downstream C-terminal segment, including residues 136–154. Domain boundaries are based on previously reported structural and functional characterizations of HBx. This schematic is intended to contextualize the spatial distribution of variants rather than infer functional effects. Mapping of the detected mutations onto the linear structure of HBx revealed that the variants were distributed across several known functional regions of the protein (Fig. 4). Four substitutions (S19A, K23R, P38T, and R47Q) occurred within the N-terminal regulatory region, a segment previously associated with transcriptional modulation. Additional mutations (T55I, C61S, and C69R) localized to the proximal transactivation and mitochondrial-targeting region, which has been implicated in HBx-mediated alterations of cellular signaling. Several variants (V88A, K94E, A100T) mapped to the H-box/DDB1-binding motif, a domain essential for HBx interaction with the host DDB1–CUL4 complex. Substitutions D102N, L113P, Q116R, E122K, and A126T were positioned within or adjacent to the BH3-like region, which is thought to participate in the modulation of apoptotic pathways. The most notable finding was the novel G135* nonsense mutation, which introduces a premature stop codon and truncates the C-terminal portion of HBx, resulting in the loss of residues 136–154. Since the C-terminus contains regulatory elements involved in protein stability and host-factor interactions, this observation may be relevant to HBx function, although its biological implications require experimental validation.

**Fig. 5:**
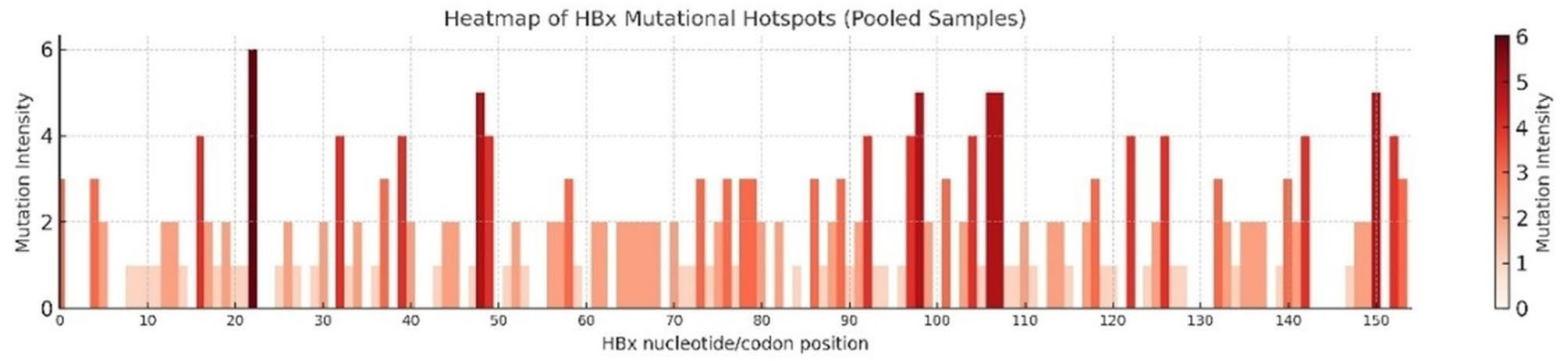
Heatmap of HBx mutational hotspots in pooled samples

The distribution and frequency of nucleotide substitutions in HBx are shown in Fig. 4. Each bar represents a nucleotide/codon position, with color intensity indicating mutation frequency. Several high-intensity clusters were observed, particularly in the C-terminal domain, suggesting a functional relevance in HBV pathogenesis and hepatocarcinogenesis. Heatmap analysis of the pooled HBx sequences revealed widespread mutational heterogeneity throughout the gene (Fig. 5). Multiple mutational hotspots were detected, with the most prominent clusters being localized within the C-terminal region. These high-intensity mutations overlap with functionally important domains of HBx, indicating their potential contributions to viral persistence, immune evasion, and hepatocarcinogenesis.

### Phylogenetic analysis

**Fig. 6:**
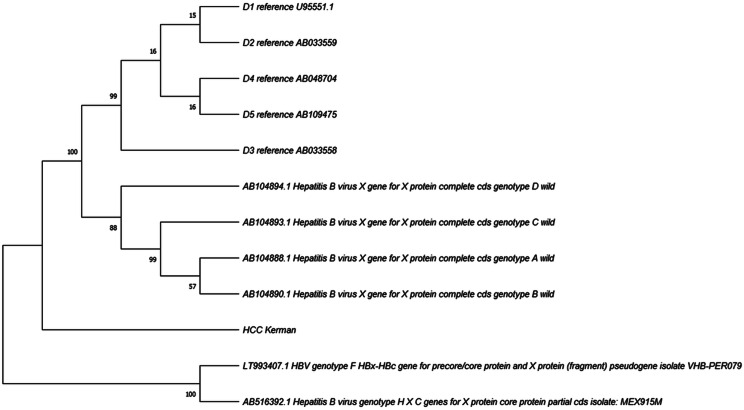
Neighbor-joining phylogenetic tree of HBx sequences showing the pooled patient-derived consensus clustering within genotype D1. Bootstrap values (1000 replicates) are indicated at major nodes; scale bar represents nucleotide substitutions per site

Phylogenetic reconstruction of the HBx consensus sequence obtained from pooled HCC samples and reference HBV strains confirmed clustering within genotype D1, which is the predominant subgenotype in Iran. The patient-derived sequences grouped tightly with D1 references, showing high bootstrap support (> 90%) and no evidence of admixture with non-D lineages (Fig. 6). The relatively short branch length indicates that the detected variants represent intra-genotypic divergence, rather than inter-genotypic recombination. Importantly, a novel truncation (G135*) and several amino acid substitutions in the H-box and BH3-like motifs were mapped within this D1 lineage, suggesting that these alterations are region-specific adaptive events rather than genotype shifts. This highlights the evolutionary stability of genotype D1 in Iran while emphasizing the clinical significance of intra-genotypic variability in driving hepatocarcinogenesis.

### 3D structural modeling of HBx in reference and HCC-derived variants

**Fig. 7:**
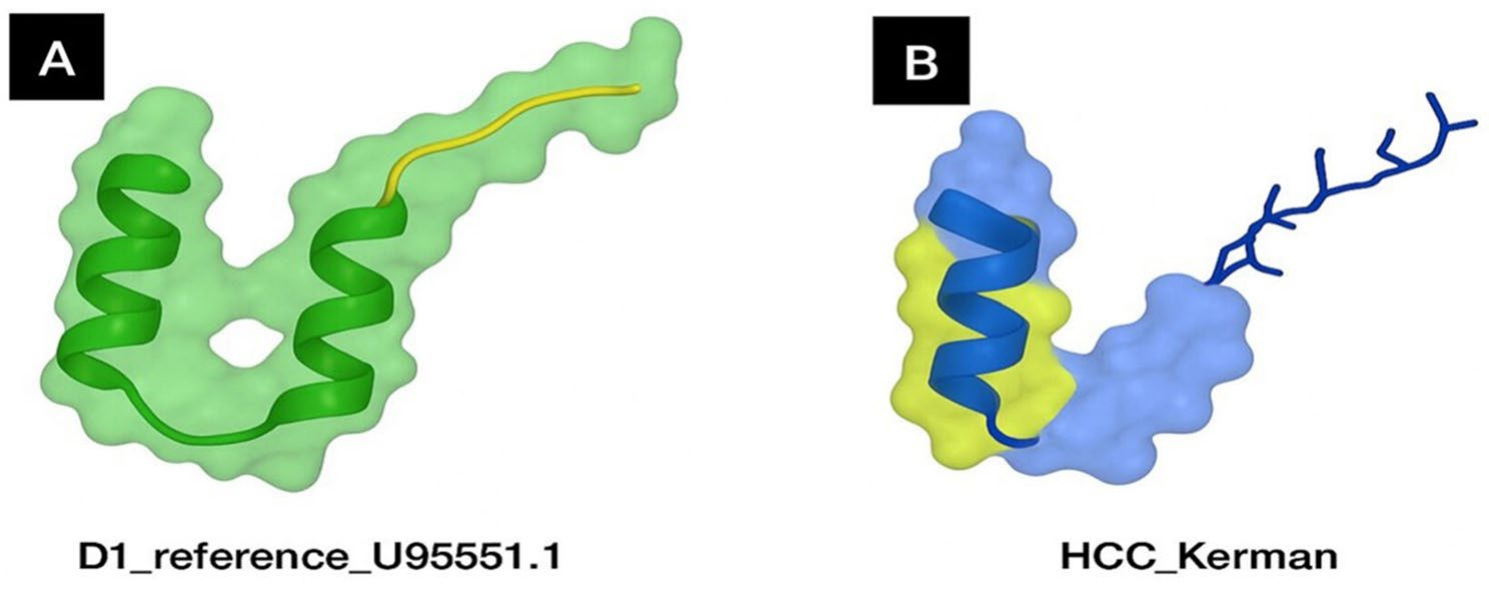
Comparative 3D structural modeling of the HBx protein in reference and patient-derived strains

Predicted tertiary structures (Fig. 7) are shown for (A) the HBx reference protein (GenBank accession number: U95551.1) and (B) an HBx variant obtained from pooled serum samples of patients with HCC from Kerman, Iran. Surface and ribbon representations highlight the overall conformational differences between reference and HCC-derived proteins. Color coding: Reference HBx in green/yellow- and HCC mutant in blue/yellow. To explore the structural basis of HBx variability in hepatocarcinogenesis, we compared the predicted tertiary structure of the reference HBx protein (GenBank accession number: U95551.1) with that of a variant sequence obtained from pooled serum samples of patients wit HCC in Kerman, Iran. The reference HBx model exhibited two conserved α-helices connected by a flexible loop, maintaining a spatial arrangement consistent with its role in transcriptional regulation and signaling. In contrast, the HCC-derived variant displayed an altered helical orientation and surface topography, indicating a shift in protein compactness and solvent accessibility. Such modifications are predicted to affect the interaction interfaces of HBx with host regulatory proteins and transcription factors. Given the established role of HBx in modulating cell proliferation, apoptosis, and DNA repair, these structural perturbations may reprogram HBx’s interactome of HBx, thereby enhancing its oncogenic potential in hepatocytes. Importantly, the observed conformational divergence in the HCC-pooled variant supports the hypothesis that specific HBx structural states contribute to liver tumorigenesis in distinct geographical and clinical contexts.

### HBx interactome analysis

**Fig. 8:**
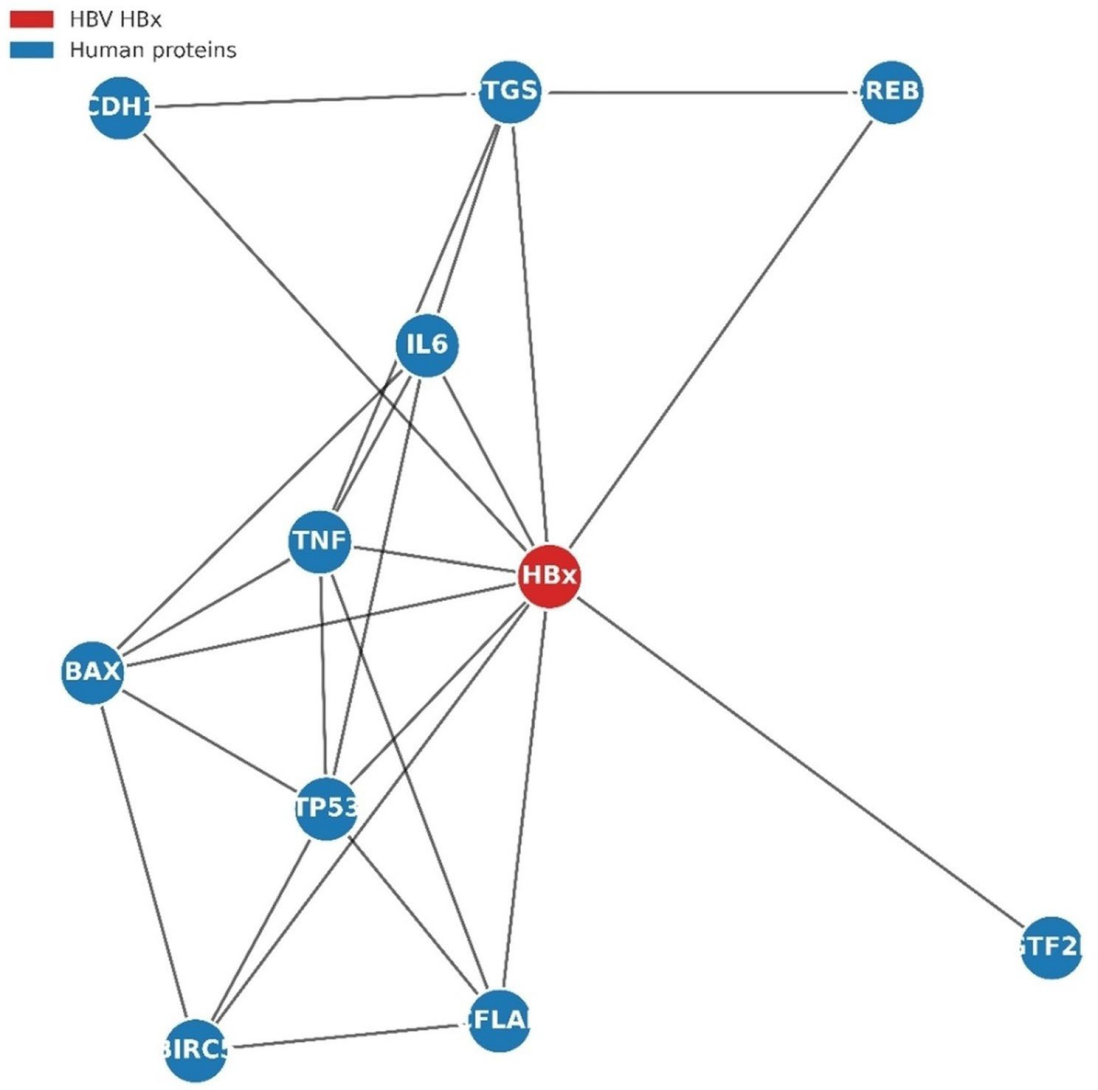
Protein–protein interaction (PPI) network of HBx and host proteins

STRING database analysis identified multiple host proteins that interact with HBx (red nodes). Edges represent experimentally validated or predicted functional associations, with line thickness proportional to the confidence score. HBx interacts with key regulators of apoptosis (TP53, BAX, BIRC5, and CFLAR), inflammation (IL6, TNF, and PTGS2), transcriptional control (CREB1 and- GTF2B), and cell adhesion (CDH1). This finding suggests that HBx modulates diverse cellular pathways to promote viral persistence and hepatocarcinogenesis. Protein–protein interaction (PPI) analysis using the STRING database revealed a highly connected network centered on the HBx, indicating its central role as a viral hub protein (Fig. 8). HBx strongly predicted and experimentally supported interactions with multiple host proteins that are critically involved in apoptosis, inflammation, transcriptional regulation, and cell signaling. Among the key tumor suppressors, TP53 and BAX are directly linked to HBx, suggesting that HBx may modulate apoptotic signaling and impair p53-mediated tumor suppression. HBx is also associated with CFLAR and BIRC5, both of which regulate apoptotic cascades and promote cell survival, highlighting a potential mechanism for viral persistence and oncogenesis. In addition, HBx interacts with several pro-inflammatory mediators, including TNF, IL6, and PTGS2, underscoring its contribution to chronic hepatic inflammation. Persistent activation of these cytokine pathways is a well-established driver of hepatic fibrosis and HCC. Interactions with CREB1 and GTF2B, both transcriptional regulators, further suggested that HBx may exert wide-ranging effects on host gene expression, thereby reprogramming cellular transcriptional responses to favor viral replication. Moreover, the identified link with CDH1, a central adhesion molecule, points toward a potential role of HBx in modulating epithelial integrity and facilitating processes such as epithelial–mesenchymal transition (EMT), a key event in HCC progression. Overall, this PPI network emphasizes the pleiotropic role of HBx in altering the host cellular pathways. By simultaneously engaging apoptotic regulators, inflammatory cytokines, transcription factors, and adhesion molecules, HBx appears to orchestrate a multi-layered disruption of host cell homeostasis. These findings provide mechanistic insights into how HBx contributes to viral persistence, immune evasion, and ultimately, the pathogenesis of HCC.

## Discussion

### Principal findings

In this study, pooled HRM analysis and sequencing identified multiple HBx mutations in HCC patients with chronic HBV infection, clustering within the H-box, BH3-like motif, zinc-binding CCCH domain, and C-terminal region. These domains are central to HBx function, mediating interactions with host factors, apoptosis regulation, and viral transcription. Importantly, we observed a novel nonsense mutation at codon 135 (G135*), truncating the last 20 amino acids of HBx, which has not been previously reported in an Iranian cohort. These findings highlight potential mechanistic links between viral genetics and hepatocarcinogenesis, warranting further functional investigation [1, 2].

### Clinical correlations and host relevance

The pooled clinical dataset from Kerman patients suggested a trend toward higher HBV viral load and SGOT activity among HBeAg-positive cases, consistent with enhanced viral replication and hepatocellular injury. While these associations are observational, they may reflect underlying effects of HBx mutations. For example, truncations in the C-terminal tail (e.g., G135*) and variants in the H-box (V88A, K94E, A100T) or BH3-like motif (L113P, Q116R, E122K, A126T) may potentially influence hepatocyte proliferation, apoptosis regulation, and viral persistence, as predicted by prior studies and in silico analyses [5–8]. These interpretations, however, require experimental validation to establish causal relationships.

### HBx structural motifs and functional implications

The mutational pattern identified across the HBx sequence in this study spans several regions previously implicated in transcriptional regulation, host–virus interactions, and oncogenic signaling. Variants detected in the N-terminal regulatory region fall within a segment known to influence early transcription-modulatory properties of HBx [9]. Although the functional significance of the specific substitutions observed here (S19A, K23R, P38T, R47Q) cannot be inferred without experimental validation, their location within regulatory elements suggests they may subtly modulate HBx-mediated host responses. Mutations situated within the transactivation region and the proximal mitochondrial interaction interface (T55I, C61S, C69R) overlap with domains previously shown to contribute to HBx-driven transcriptional activation, mitochondrial localization, and perturbation of cellular signaling pathways [10]. However, as our study is limited to sequence and structural modeling, any potential impact of these substitutions should be interpreted cautiously. Several substitutions mapped to or near the H-box (DDB1-binding domain), including V88A, K94E, and A100T. This motif is essential for HBx recruitment of DDB1–CUL4, a host complex involved in epigenetic regulation and viral replication [11]. Changes within this region may influence host-factor interaction surfaces, although targeted biochemical assays would be required to test such hypotheses. A cluster of variants within the p53-interaction region (residues ~ 102–136) was also observed. Prior studies have reported that this segment participates in modulating p53-dependent transcription and apoptosis [12]. While substitutions such as D102N, L113P, Q116R, E122K, and A126T fall within this zone, the structural consequences derived from modeling remain speculative and require in-vitro studies for confirmation. The most notable finding is the novel premature stop-gain mutation G135*, which results in the loss of the downstream C-terminal segment, including residues essential for regulatory interactions and protein stability. C-terminal truncations of HBx have frequently been reported in HBV-related HCC and are thought to alter oncogenic signaling, mitochondrial localization, and proteasomal regulation [13, 14]. Importantly, the functional effects of truncations depend strongly on the exact position of the stop codon. Therefore, while the structural loss caused by G135* is clear, its phenotypic consequences remain uncertain without experimental validation. Overall, these findings contribute to the understanding of HBx sequence variability in genotype D1 and provide a structural map of mutation placement within functionally important regions. However, the biological implications of these sequence alterations remain hypothetical, emphasizing the need for mechanistic and cell-based studies to determine whether the identified mutations—including G135*—play any role in hepatocarcinogenesis.

#### STRING network and pathway perturbations

Protein–protein interaction mapping indicated that HBx is a hub protein interacting with apoptosis regulators (TP53, BAX, BIRC5), pro-inflammatory mediators (TNF, IL6), and transcriptional modulators (CREB1, GTF2B). Variants disrupting the H-box and C-terminal domains were predicted to alter HBx connectivity within these networks, potentially amplifying NF-κB, Wnt/β-catenin, and MAPK signaling, which may contribute to persistent inflammation, immune evasion, and hepatocyte transformation [13, 15]. The C-terminal truncation (G135*) may influence HBx proteostasis by disrupting hydrophobic interactions that stabilize the native fold, potentially enhancing susceptibility to proteasomal degradation [16].

### Potential impact on HBx interactome

HBx functions as a dynamic hub, engaging in multiple interactions with viral proteins (e.g., HBc, HBs) and host factors (e.g., DDB1, p53, CREB1), collectively regulating viral replication, transcription, and cell survival [17]. The identified mutations, particularly the C-terminal truncation (G135*) and H-box/BH3-like variants, are predicted to perturb this interactomic network. Truncation of the C-terminal tail may abolish critical binding interfaces, leading to reduced association with specific host cofactors and altered subcellular localization. Similarly, H-box and BH3-like mutations could weaken or modify interactions with DDB1 and Bcl-2 family proteins, respectively, potentially reshaping downstream signaling pathways such as NF-κB, MAPK, and Wnt/β-catenin [13, 15]. Although experimental validation is required, these findings suggest that HBx mutational landscapes may dynamically rewire protein–protein networks, influencing viral fitness, host cell survival, and oncogenic potential in HCC.

#### Phylogenetic positioning

Phylogenetic reconstruction anchored our pooled HBx consensus within genotype D1, the dominant subgenotype in Iran [18, 19]. The absence of admixture with non-D genotypes suggests that the observed variants are intra-genotypic adaptations. This supports their novelty within genotype D1 and indicates potential region-specific mutational pressures linked to HCC development.

#### Overlap with the polymerase ORF (Pol) & therapeutic relevance

Because of the overlapping reading frames of HBx and the polymerase gene, nucleotide substitutions in HBx may inadvertently affect polymerase structure and replication efficiency. Previous studies have demonstrated that changes in this region can alter replication fitness, polymerase activity, and antiviral drug sensitivity [20–22]. Thus, our identified variants may exert dual effects, disrupting HBx regulatory functions while modulating polymerase biology, with potential clinical implications. Truncating mutations, such as G135*, as well as variants within the H-box and BH3-like motifs, are predicted to enhance hepatocyte survival, confer resistance to apoptosis, and amplify oncogenic signaling [1, 2, 5–8, 23]. Experimental validation is required to confirm these effects.

#### Novelty and contribution

The most striking novel finding is the G135* nonsense mutation, resulting in the loss of terminal 20 residues, including part of the zinc-binding motif. Although C-terminal truncations of HBx have been linked to HCC in Asian cohorts [1, 23], to our knowledge, this specific variant has not been previously reported in Iranian populations. Its enrichment in our pooled HCC sample suggests a potential regional hotspot that warrants further investigation. Together with variants in conserved domains, this supports the concept of HBx-driven hepatocarcinogenesis through both the loss of tumor suppressor interactions and gain of oncogenic properties.

### Limitations

The pooled sequencing approach allowed efficient detection of shared HBx mutations, including the novel G135* truncation. However, this study is primarily observational, and functional consequences of these mutations on hepatocyte proliferation, migration, apoptosis, or therapeutic response were not experimentally evaluated. Future studies using individual-sample sequencing and in vitro functional assays are warranted to validate the oncogenic potential and mechanistic effects of these variants.

## Conclusions

These HBx variants, including the novel C-terminal truncation, are clustered within functionally important domains and may be associated with viral persistence and hepatocarcinogenesis in HCC. While these observations are based on sequencing and in silico analyses, experimental validation is required to establish their functional impact and potential as biomarkers or therapeutic targets.

## Data Availability

The datasets generated and/or analyzed during the current study are available in GenBank under the accession number: MN120773.1 (https://www.ncbi.nlm.nih.gov/nuccore/). Additional datasets supporting the findings of this study are available from the corresponding author upon request.
